# Death by massive air sac fluke (Trematoda: *Bothriogaster variolaris*) infection in a free-ranging snail kite (*Rostrhamus sociabilis*)

**DOI:** 10.1016/j.ijppaw.2022.09.001

**Published:** 2022-09-08

**Authors:** Eduardo A. Díaz, Gustavo Donoso, Juan D. Mosquera, Darío X. Ramírez-Villacís, Gerardo González, Sonia Zapata, Diego F. Cisneros-Heredia

**Affiliations:** a Universidad San Francisco de Quito USFQ, Escuela de Medicina Veterinaria, Instituto de Biodiversidad Tropical IBIOTROP, Hospital de Fauna Silvestre TUERI, Quito, Ecuador; b Universidad San Francisco de Quito USFQ, Colegio de Ciencias Biológicas y Ambientales COCIBA, Instituto de Microbiología, Quito, Ecuador; c Universidad San Francisco de Quito USFQ, Colegio de Ciencias e Ingenierías, Ingeniería en Agronomía, Laboratorio de Biotecnología Agrícola y de Alimentos, Quito, Ecuador; d Universidad San Francisco de Quito USFQ, Colegio de Ciencias Biológicas y Ambientales COCIBA, Instituto de Biodiversidad Tropical IBIOTROP, Laboratorio de Zoología Terrestre, Museo de Zoología, Quito, 170901, Ecuador

**Keywords:** Cyclocoelidae, Parasitosis, Pneumatic bones, Bird of prey, Airways

## Abstract

Helminths are not usually considered important pathogens for birds of prey. There is a single published report of mortality in raptors due to an air sac trematode infection. We report a well-documented death case from massive infection by an air sac trematode of the family Cyclocoelidae in a wild-caught, juvenile male Snail Kite (*Rostrhamus sociabilis*) in Ecuador. The necropsy of a Snail Kite revealed more than 200 trematodes among air sacs, lungs, heart, gizzard, proventriculus, and liver. Within air sacs and lungs, mature flukes were associated with sacculitis, bronchitis, pneumonia, and atelectasis. Using an integrative taxonomic approach with morphological and molecular data, we identified the parasites as *Bothrigaster variolaris* (Trematoda: Cyclocoelidae: Ophthalmophaginae). This case provides the first evidence for the pathologic presence of air sac trematodes associated with morbidity in birds of prey in South America. Our results suggest that cyclocoelids may cause debilitation and significant clinical lesions in birds of prey, with potentially fatal consequences.

## Highlights

• Necropsy revealed massive air sac infection caused by fluckes in a deceased Snail Kite.
• The trematode *Bothrigaster variolaris* was identified using morphological and molecular data.
• Snail Kite death was likely caused by parasite bronchial obstruction and associated lesions.
• This is the first report of air sac trematodes as a cause of death in birds of prey in South America.
• Trematode infection may cause significant clinical lesions and death in birds of prey.

## Graphical abstract

**Figure:**
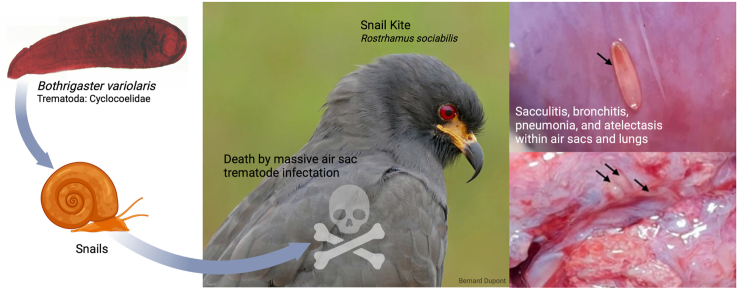

## 1 Introduction

A wide variety of helminths infect birds of prey, including roundworms (nematodes), flukes (trematodes), tapeworms (cestodes), and spiny-headed worms (acanthocephalans) (Ward, 1975; Redig, 1993; Krone and Cooper, 2002; Atkinson et al., 2008). Although helminth infections cause morbidity and mortality in captive and wild birds of prey, helminths are not usually considered important pathogens (Krone and Cooper, 2002; Krone, 2007). However, few studies have dealt with the pathogenicity and impacts of helminths in wild populations of birds of prey (Ward, 1975; Redig, 1993; Smith, 1993, 1996; Lacina and Bird, 2000; Krone and Cooper, 2002; Krone, 2007; Atkinson et al., 2008; Coulson et al., 2010; Santos et al., 2010; Andery et al., 2013; Vasilevytch et al., 2016).

Death associated with infections of air sac trematodes has been described in several captive and wild species of birds (Cole et al., 1995; Dronen et al., 2009; Libert et al., 2012; Delaski et al., 2015; Galosi et al., 2019). To the best of our knowledge, there is only one report of death by air sac trematodes in raptors, specifically in Snail Kites *Rostrhamus sociabilis* (Accipitridae) (Cole et al., 1995). Snail Kites are a bird of prey associated with open freshwater wetlands across America, from Florida (USA), Cuba and eastern México to northeastern Argentina and Uruguay (Bierregaard, Jr. and Kirwan, 2013). This kite is specialised in feeding on apple snails (*Pomacea* spp.) (Beissinger, 1990; Ferguson-Lees and Christie, 2001).

Despite the high diversity of birds of prey in Ecuador (49 spp. of Accipitriformes, 6 Cathartiformes, 19 Falconiformes, 29 Strigiformes), information on helminths in Ecuadorian birds of prey is minimal (Petri, 1942; Van Cleave, 1940). In the present study, we describe a death from massive air sac trematodiasis in a Snail Kite, the first report for South America.

## 2 Materials and methods

A free-ranging juvenile male Snail Kite was brought from Daule (1°51′48.05″ S 79°58′41.12″ W, 5 m above sea level), province of Guayas, Ecuador, on January 07, 2020 to the *Hospital de Fauna Silvestre TUERI*, a wildlife hospital dedicated to the rescue, medical treatment, and rehabilitation of wild animals in Ecuador. The hospital is in the town of Cumbayá, Quito Metropolitan District, Ecuador, and part of the Institute of Tropical Biodiversity IBIOTROP of Universidad San Francisco de Quito USFQ. Upon arrival, the snail kite showed signs of shock, prostration, subdued mental status, and hypothermia, with severe dehydration and dyspnea. Warm fluids were administered, and it was placed in an oxygen chamber to stabilise it. The animal died 6 h after hospital admission, and a complete necropsy was performed immediately. Tissue samples from all organs were collected and fixed in 10% buffered formalin, embedded in paraffin, and stained with hematoxylin and eosin (H&E) for histopathology, using the protocol described by Slaoui and Fiette (2011). Thirty-five flukes were collected upon post-mortem examination and fixed in 96% ethanol for molecular analysis. Specimens were stained with hydrochloric carmine for morphological studies and mounted in Eukit medium. Taxonomic identification was based on Dronen and Blend (2015). Images of mounted parasites were taken with an AmScope 18 MP USB 3.0 color CMOS C-mount microscope camera (magnification 4×) coupled with a Zeiss V20 microscope. Graphical abstract was created with Biorender.com.

DNA was extracted from preserved trematodes with PureLink™ Genomic DNA Mini Kit (ThermoFisher, US) following manufacturer's instructions. For polymerase chain reaction (PCR), we used primers C–F 5′-ATGGCTCATTAAATCAGCTAT-3′ and A-R 5′-TGCTTTGAGCACTCAAATTTG-3′ targeting nuclear 18s rDNA (Routtu et al., 2014). PCR mix was prepared with Platinum™ Taq DNA Polymerase (ThermoFisher, US) and concentrations of reagents per reaction were: 1X Buffer, 1.5 mM MgCl_2_, 0.2 mM dNTPs, 0.6 μM of each primer and 0.5 U of Taq DNA polymerase. PCR was performed in an Applied Biosystems SimpliAmp thermal cycler (ThermoFisher, US) with cycling conditions: Initial denaturation at 94 °C for 2 min, followed by 35 cycles of denaturation at 94 °C for 15 s, annealing at 60 °C for 2 min and extension at 72 °C for 2 min. The final extension was carried at 72 °C for 1 min. PCR products were visualised in 1.5% agarose gel, lyophilised, and send for purification and sequencing at Macrogen, Korea. A consensus sequence was submitted to GenBank with accession number MT271792. The 18s rDNA sequences of taxa from each family in the superfamily Echinostomatoidea, and outgroups (Schistosomatoidea and Opisthorchioidea) were obtained from Pérez-Ponce de León and Hernández-Mena (2019). Additional accessions from family Cycloelidae available at NCBI were included (LC520232, KU877902, KU877901 and KU877900). Alignments were built in MEGAX using ClustalW with default parameters (Kumar et al., 2018). A phylogenetic tree was constructed using Bayesian Inference (BI) with nucleotide evolution model GTR + GAMMA + I (MCMC 40 million generations) in Beast v1.10.4 (Drummond and Rambaut, 2007). A consensus tree was obtained with maximum clade credibility (burning = 1500 trees) in TreeAnnotator v1.10.4 and rendered in FigTree v1.4.4 (http://tree.bio.ed.ac.uk/software/figtree/, by Andrew Rambaut).

## 3 Results

**Fig. 1:**
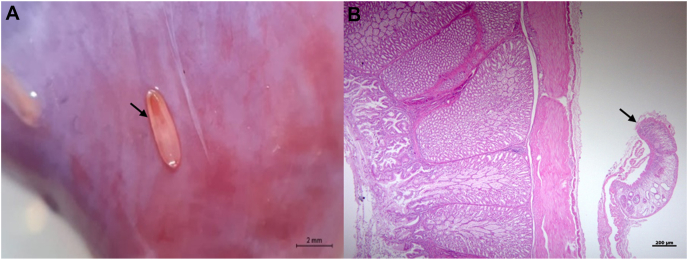
Trematodes in serosa of heart and proventriculus of Snail Kite (*Rostrhamus sociabilis*): A. Close up image of a trematode (arrow) in the pericardium during necropsy. B. Histologic capture of a trematode (arrow) present in the serosa of the proventriculus (H&E).

**Fig. 2:**
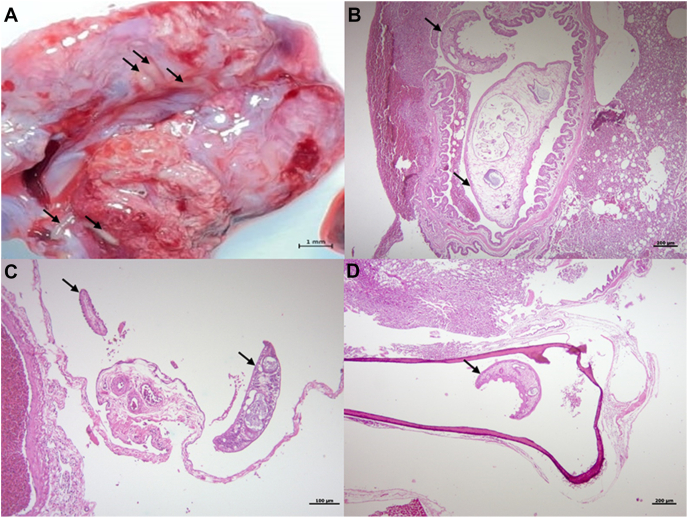
Trematodes in respiratory system and pneumatic bone of a Snail Kite (*Rostrhamus sociabilis*): A. Macroscopic image of the lungs, with presence of trematodes (arrows) found during necropsy. B. Trematodes (arrows) colonising the lumen of a tertiary bronchus (10x H&E) C. Trematodes (arrows) in an abdominal air sac (10x H&E) D. A parasite (arrow) in the coracoid bone (10x H&E).

**Fig. 3:**
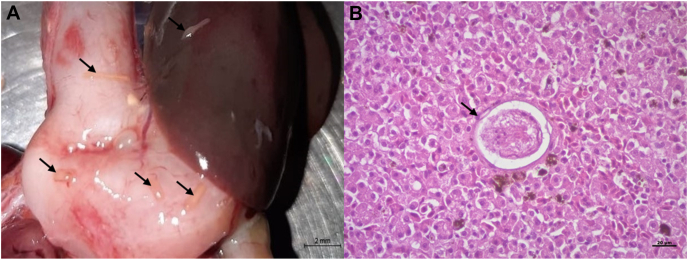
Trematodes in the liver of a Snail Kite (*Rostrhamus sociabilis*): A. Trematodes (arrows) in the serosa of proventriculus, gizzard and liver found during postmortem procedure. B. Miracidium (arrow) in the liver parenchyma (40x H&E).

**Fig. 4:**
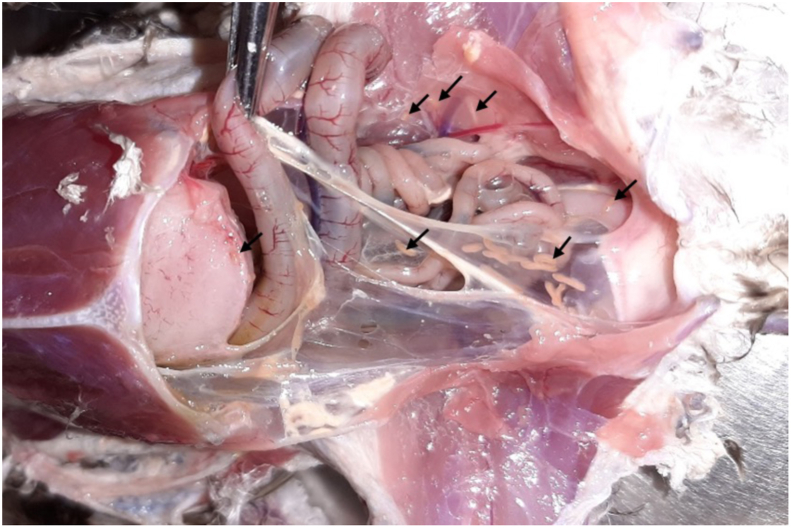
Trematodes in the right abdominal air sac, serosa of gastrointestinal tract and celomic cavity of a Snail Kite (*Rostrhamus sociabilis*).

The snail kite presented poor body condition on gross external examination, but no significant disorders were detected other than minor contusions. During the internal inspection, trematodes were observed in abundance over the pericardium (Fig. 1A), epicardium, thoracic and abdominal air sacs, lungs (Fig. 2A), gizzard, proventriculus and liver (Fig. 3A). Significant macroscopic changes in the respiratory system were observed, including thickened-opaque air sacs and lung parenchyma with multifocal coalescing dark-red firm patches with depressed surfaces. At lung dissection, congestion and parasites were also observed. More than 200 flukes were counted in the entire animal (Fig. 4).

Histopathology revealed trematodes in the serosa of the proventriculus (Fig. 1B), bronchi (Fig. 2B) and air sacs (Fig. 2C). A mature trematode was discovered in the coracoid bone (Fig. 2D)—other bones were not examined, and multiple miracidia were in the liver parenchyma (Fig. 3B). In the lungs, parasites were located within the lumen of primary and secondary bronchi and parabronchi; haemorrhage was observed in the lumen of bronchi and parabronchi and surrounding tissues where parasites were showed signs of congestion and atelectasis. Lung parenchyma presented perivascular lymphocytic pneumonia and bronchitis with loss of ciliated epithelium and hyperplasia in primary bronchi. Air sacs had lymphoplasmacytic and heterophil airsacculitis accompanied by fibrosis and oedema. The liver showed multifocal granulomatous and heterophilic hepatitis with scattered trematode larval structures.

**Fig. 5:**
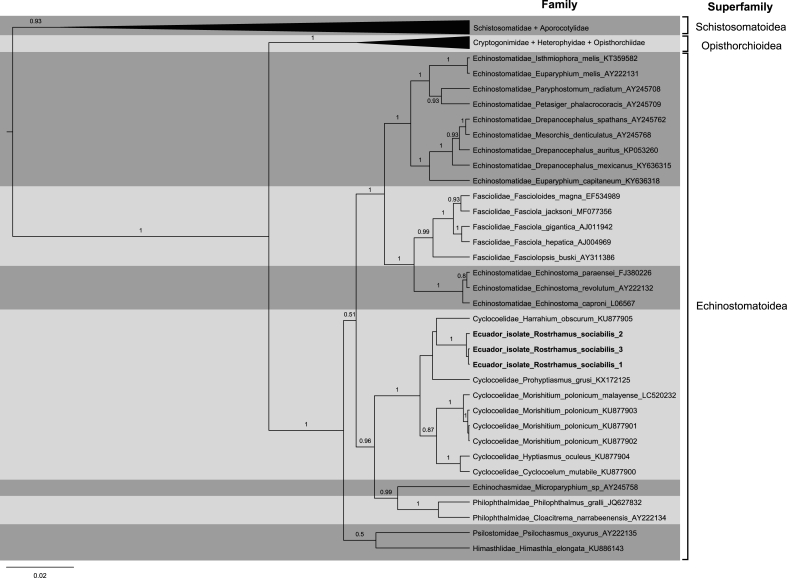
Bayesian inference phylogenetic tree of the Echinostomatoidea superfamily showing the position of trematodes extracted from Snail Kite from Ecuador (in bold). The tree was built using small subunit of the ribosomal RNA gene (18 S rDNA) in Beast v1.10.4. Bayesian posterior probability values ≥ 0.5 are shown in branches. Family, genus, and species from each sequence are listed along with their accession number. Family delimitations are indicated with grey boxes. Sequences of Schinostomatoidea and Opisthorchioidea were used as outgroups. Scale bar indicates number of expected substitutions per site.

Bayesian analyses of 18s rDNA revealed that these trematodes are a distinct clade within the superfamily Echinostomatoidea, family Cyclocoelidae (Fig. 5). We included in our analyses all available sequences from cyclocoelids in the NCBI database (72 accessions, representing ten genera and 11 species). Trematodes reported in this study form a clade with *Harrahium obscurum* and *Prohyptiasmus grusi*, but the posterior probability is less than 0.5, indicating that its phylogenic relationships within the family are unresolved.

**Fig. 6:**
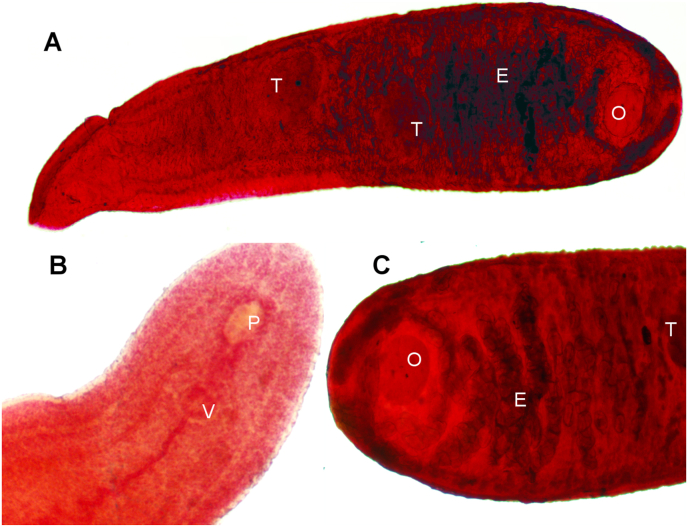
Full body (A), anterior end (B) and posterior end (C) of trematode *Bothrigaster variolaris* from a Snail Kite (*Rostrhamus sociabilis*). T = midbody testes, V = ventral sucker, p = pharynx, O = postesticular ovary, E = eggs.

Morphological characters confirm the identification of these trematodes as members of the family Cyclocoelidae. Specimens showed a lanceolate body (width = 0.825 mm, length = 2.992 mm), with a ventral sucker (Fig. 6A) and tapered anterior and rounded posterior ends (Fig. 6B). The two midbody testes (0.312 × 0.237 mm and 0.326 × 0.223 mm) formed a nearly straight line with a postesticular ovary (0.242 × 0.158 mm), and eggs measured 0.059 × 0.038 mm (Fig. 6C). The presence of the ventral sucker is characteristic of the genus *Bothrigaster* (subfamily Ophthalmophaginae), which currently has only one species, *B. variolaris*.

## 4 Discussion and conclusions

Our results suggest the Snail Kite likely died of suffocation due to bronchial obstruction by parasites and respiratory tissue lesions. Death from air sacs trematodiasis has usually been reported in captive passerine birds, and Passeriformes have been traditionally considered to have a greater risk of parasitosis than other bird clades (Dronen et al., 2006a, 2009; Libert et al., 2012; Delaski et al., 2015; Galosi et al., 2019). However, our report coincides with Cole et al. (1995), who described a massive trematode infection as possible cause of death in two Snail Kites (a fledgling and a subadult) from Florida. Those birds had opaque air sacs and tan granular deposits accumulated in folds and angles of tissues, with moderate pyogranulomatous bronchitis and peribronchitis, mild squamous metaplasia of the epithelium near intrabronchial trematodes, mild granulomatous airsaculitis, and the fledgling showed many trematodes. Cole et al. (1995) morphologically identified the trematodes involved in both deaths as *B. variolaris* (Digenea: Cyclocoelidae), a cyclocoelid reported as a parasite of Snail Kites in USA, Brazil, Cuba, and Argentina (Cole et al., 1995; Drago et al., 2014; Drago and Lunaschi, 2015; Dronen and Blend, 2015).

The presence of a mature trematode in the coracoid bone has been described as an atypical parasitic migration within the avian airways system, leading to irreversible damage of the host skeleton (Hawkins et al., 2001). The fluke possibly entered the coracoid bone through the connections between the clavicular air sacs and pneumatic bones. Avian air sacs have several diverticula in the pneumatic bones, i.e., the clavicular sac connected to the lungs via the first and second medioventral bronchi (Orhan et al., 2009).

Trematodes of the family Cyclocoelidae are large-bodied flukes found as adults in the body cavity, air sacs, lungs, nasal and infraorbital sinuses, or hypothalamus of birds, and occasionally in mammals (Dronen, 2007a; Dronen et al., 2009; Dronen and Blend, 2015). The family Cyclocoelidae includes 22 genera and ca. 128 species (Dronen and Blend, 2015). However, only half of the genera and less than 10% of species have molecular information available. Species identification of trematodes based solely on morphology may prove inaccurate because of few autapomorphic features and physical distortion of specimens during mounting (Sitko et al., 2017). Enrichment of databases with novel cyclocoelid sequences and evaluation of morphological characters will aid in better resolving phylogenetic relationships within Cyclocoelidae. Cyclocoelids are cosmopolitan parasites primarily infecting wetland birds. The complete life cycle of most species in the Cyclocoelidae family has not been fully elucidated. When known, they include either freshwater or terrestrial snails as their first intermediate host of larvae stages and birds as definitive vertebrate hosts of adults (Taft, 1971, 1973, 1975, 1986; Conti et al., 1985; McLaughlin, 1983, 1986; Dronen, 2007a, 2007b; Dronen et al., 2006b, 2006c, 2009; Lunaschi et al., 2007; Blend and Dronen, 2008; Dronen and Tkach, 2013; Shutler et al., 2016; Gomez-Puerta et al., 2018; López-Jiménez et al., 2018). Snail Kites most likely become infected with cyclocoelids by eating aquatic snails harbouring larval stages (Cole et al., 1995). Multiple larvae in the liver parenchyma reported in this study could indicate that after being ingested, parasitic larvae pass through liver ducts and later via hematogenous migration reach and break out of the pulmonary vasculature to invade the respiratory system.

Snail Kites are not threatened, globally or in Ecuador (BirdLife International, 2016; Freile et al., 2019), and increasing and expanding populations have been reported across its range (Ferguson-Lees and Christie, 2001; Bierregaard, Jr. and Kirwan, 2013). Ecuadorian populations may be increasing due to habitat modification to grow rice and high densities of freshwater *Pomacea* snails (Horgan et al., 2014b). However, growing snail populations are due to an invasive species, *Pomacea canaliculata*, considered a pest due to its severe impacts on agriculture, human health, and the environment (Horgan et al., 2014a). *Pomaceae* snails are known intermediate hosts of vertebrate parasites and maybe involved in the transmission of cyclocoelid trematodes (Conti et al., 1985; Lunaschi et al., 2007; Hayes et al., 2015; Gutierre et al., 2019). While the establishment and expansion of invasive snails have apparently benefited Snail Kite populations, parasites transmitted by invasive snails and its effects on snail predators, such as Snail Kite and Limpkin (*Aramus guarauna*), have yet to be explored (Rawlings et al., 2007).

## Ethics approval

This study was part of an interinstitutional cooperation agreement for access to genetic resources between the Ministry of Environment, Water and Ecological Transition of Ecuador (Ministerio del Ambiente, Agua y Transición Ecológica MAATE) and Universidad San Francisco de Quito USFQ (No. MAE-DNB-CM-2018-0085), and under a wildlife management patent issued by MAATE (No. 011-2021-FAU-OTQ-DZ2E-MAAE).

## Funding

This work was supported by operational and research funds provided by 10.13039/501100010654Universidad San Francisco de Quito USFQ for the Hospital de Fauna Silvestre TUERI, Instituto de Biodiversidad Tropical IBIOTROP.

## Authors' contributions

GD, GG, and EAD dealt with clinical case and sample collection; EAD and GD reviewed and validated histopathological data; JDM, DXRV, and SZ worked on the morphological and molecular analyses; DFCH and EAD supervised work at the hospital; EAD, JDM and DFCH prepared figures and data visualisation; DFCH and SZ oversaw project administration and funding acquisition; SZ and DFCH provided hospital and lab resources; DFCH wrote the original draft; DFCH and EAD reviewed and edited the manuscript. All authors reviewed and approved the final manuscript.

## Availability of data and materials

All data generated or analysed during this study are included in this published article.

## Declaration of competing interest

The authors declare that they have no competing interests.
